# Polish immigrants’ access to colorectal cancer screening in Norway – a qualitative study

**DOI:** 10.1186/s12913-022-08719-3

**Published:** 2022-11-10

**Authors:** Sameer Bhargava, Elżbieta Czapka, Solveig Hofvind, Maria Kristiansen, Esperanza Diaz, Paula Berstad

**Affiliations:** 1 Cancer Registry of Norway, P.O. Box 5313, N-0304 Majorstuen, Oslo, Norway; 2 Department of Medicine, Division of Oncology, Bærum Hospital, Vestre Viken Hospital Trust, Bærum, Norway; 3 Department of Oncology, Akershus University Hospital, Lørenskog, Norway; 4 Sociology Institute, University of Gdansk, Gdansk, Poland; 5 Department of Health and Care Sciences, Faculty of Health Sciences, The Arctic University of Norway, Tromsø, Norway; 6 Department of Public Health & Center for Healthy Aging, Faculty of Health and Medical Sciences, University of Copenhagen, Oester Farimagsgade 5, 1353 Copenhagen, Denmark; 7 Department of Global Public Health and Primary Care, University of Bergen, Årstadveien 17, 5009 Bergen, Norway; 8 Unit for Migration and Health, Norwegian Institute of Public Health, 222 Skøyen, 0213 Oslo, Norway

**Keywords:** Emigrants and immigrants, Minority health, Colorectal cancer, Screening

## Abstract

**Background:**

The Norwegian colorectal cancer (CRC) screening programme started in May 2022. Inequities in uptake of CRC screening is a concern, and we expect that immigrants are at risk of non-uptake. Immigrants from Poland are the most populous immigrant group in Norway. The purpose of this study was to identify and explore factors that may facilitate Polish immigrants’ access to the Norwegian CRC screening programme.

**Material and methods:**

This study was based on qualitative interviews with ten Polish immigrants in Norway. The participants represented a convenience sample that varied in terms of gender, education, employment, time in Norway, place of residence, Norwegian language skills and ties to the Norwegian-Polish community. We performed thematic content analysis to understand CRC screening from the perspective of Polish immigrants, using transnationalism and Levesque’s conceptualization of accessibility as theoretical frameworks.

**Results:**

We grouped our findings into three themes; “understanding of CRC development and the need to access health care”, “binationalism” and “improving accessibility through information”. Within these themes, various factors influenced the participants’ accessibility to CRC screening, namely knowledge about the screening and about causes, development and prevention of the disease, language, choice of screening country, trust in health personnel’s competence, information needs, methods and sources, as well as participants’ perception of the faecal immunochemical test screening user manual. These factors were further influenced by communication between the Polish community in Norway and Poland, as well as travel between the countries.

**Conclusion:**

We identified several factors that can be targeted with an aim to increase Polish immigrants’ access to the Norwegian CRC screening programme. Effective measures could include increasing cultural competence among health care providers and providing information in Polish through Polish-speaking health care professionals, general practitioners and internet portals used by the Polish-speaking community. Focusing on accessibility in a transnational setting, our findings may be of interest for policy makers and service providers planning preventive health measures for immigrants.

## Introduction

Colorectal cancer (CRC) is one of the most common cancers worldwide, and a major source of cancer-related morbidity and mortality [1]. In Norway, CRC is the second most common cancer, and the cancer type with the second highest cancer mortality [2]. CRC is potentially curable through surgery if detected at an early stage. If detected early, 5-year survival is about 98%. However, CRC symptoms may be unspecific, and may appear at an advanced stage, where surgery is no longer possible. If detected at an advanced stage with distant metastasis, less than 20% are shown to be alive five years after diagnosis [2].

Cancer screening programmes aim to detect disease at an early stage in asymptomatic individuals in order to reduce disease-specific morbidity and mortality [3]. Such programmes generally offer screening to all individuals in a target group, regardless of country of birth, income, education and other socioeconomic factors. The target group is usually defined according to the age range with the highest risk of the disease. A prerequisite for cancer screening programmes to have the intended effect is that people who receive an offer for screening take part in the screening examination as well as in follow-up of positive results.

A CRC screening pilot study was conducted in parts of Viken, the most populous county in Norway, in the period 2012–2018. Prior to this, there was no organised CRC screening in the country. A nationwide CRC screening programme started in May 2022. The programme is administered by the Cancer Registry of Norway. Participants are offered screening from the year they turn 55. Initially, five biennial rounds of faecal immunochemical test (FIT) will be the standard screening method, but once-only colonoscopy will gradually replace FIT over several years as the colonoscopy capacity in the country improves. Participation in FIT screening includes a stool sample taken at home and thereafter returning the sample to a laboratory in a prepaid envelope. If blood is detected in the sample, the participant will be offered a colonoscopy examination in a hospital in order to detect polyps, cancerous lesions and other pathology.

In 2022, about 15% of the population in Norway are immigrants, representing considerable diversity in terms of e.g. country of birth, reasons for migration, educational status, work participation and living conditions [4]. In the CRC screening pilot project, participation rate in the faecal-based screening was 46% among the invited immigrants and 60% among non-immigrants [5]. Overall, immigrant women are shown to have lower attendance than the rest of the population in the two organised, nationwide cancer screening programmes in Norway; BreastScreen Norway and CervicalScreen Norway [6, 7]. Studies from other countries show that immigrants also have lower uptake for CRC screening [8, 9], as well as for diagnostic follow-up after a positive CRC screening examination [10]. Studies from other countries have identified lack of awareness of CRC screening, lack of symptoms, questioning appropriateness and efficacy of the test, and logistical challenges as barriers to CRC screening [11]. Studies focusing on immigrants have further identified language barriers, cultural beliefs, competing life demands, low health literacy level, challenges navigating health care systems and being a recent immigrant as barriers to cancer screening [11–14]. There may be delays at each contact with the health care system, as personal factors, such as language barriers and lack of transportation, may interact with systemic barriers, such as the referral process and waiting times for diagnostics testes [15].

The Nordic welfare model aims for equity in access to health care for the entire population [16]. Norway has universal health care, which is publicly funded. All residents in Norway are entitled to medical services, regardless of age, country of birth and income. Despite health care often being referred to as “free”, patients still pay an out-of-pocket fee for services. All residents in Norway are entitled to have a general practitioner (GP), who is their first point of contact for non-acute medical issues. People with limited language skills, for instance people who don’t have Norwegian as their first language or people who use sign language, are entitled to have a public interpreter accompany them free of charge for medical appointments.

As immigrants have been shown to have lower participation in the Norwegian CRC screening pilot study and the other two organised cancer screening programmes in Norway, we expect that immigrants as a group will also have lower uptake compared with non-immigrants in the Norwegian CRC screening programme, possibly resulting in higher morbidity and mortality. If immigrants are systematically prevented from accessing screening, non-uptake can enhance health inequalities between immigrants and non-immigrants, potentially disadvantaging a large proportion of the Norwegian population.

### Polish immigrants in Norway

Immigrants from Poland are the most populous immigrant group in Norway. Over 100,000 individuals born in Poland live in Norway, accounting for thirteen percent of all immigrants and two percent of the total Norwegian population [4]. Poland’s inclusion in the European Union in 2004 saw a great increase in labour emigration from Poland, and a large proportion of Polish immigrants in Norway are labour immigrants [4]. Immigrants from Poland are also the most populous group *emigrating* from Norway [4].

Several studies have examined health-related factors among Polish immigrants in Norway. Based on data from a survey in 2016, there were no statistically significant differences between non-immigrants and Polish immigrants in terms of reporting their own health as good [17]. However, compared to non-immigrants, both Polish men and women reported more often having mental health problems and being current smokers, and less often being physically active, while Polish men more often reported sleep deprivation and being overweight, and less often having a disease. In registry-based studies of cancer incidence and stage at diagnosis among immigrants in Norway, Polish immigrants have been grouped together with other immigrants from Eastern Europe. Immigrants from Eastern Europe have been found to have lower incidence of CRC and a somewhat higher rate of advanced stage CRC than non-immigrants, but differences in stage were no longer significant after adjusting for socioeconomic factors [18, 19].

In terms of health-seeking behaviour, Polish immigrants have been found to less often seek emergency primary health care and less often have consultations for mental health problems compared to non-immigrants [20, 21]. In a survey, 18% of Polish immigrants reported that they went to a doctor in Norway when they felt ill, while 50% reported that they used to go to a doctor when feeling ill in Poland [22]. Polish immigrants have had low attendance rates in both CervicalScreen Norway and BreastScreen Norway [6, 7]. A qualitative study aiming to map out how and where immigrants from Poland and Somalia would like to receive information about cancer in general revealed that Polish immigrants would like to receive information about cancer prevention, for instance through information in Polish or from doctors with a Polish background, and that cancer was something they feared [23].

We anticipate that the most populous immigrant group in Norway is at risk of particularly low uptake of CRC screening in the organised programme in Norway. If this is true, we do not know if Polish immigrants in Norway remain unscreened, or if they undergo screening in Poland. If they remain unscreened, they will potentially be at increased risk for advanced stage disease with accompanying morbidity and mortality as compared to the majority population.

### Aim

We used qualitative interviews to identify and explore factors that may influence Polish immigrants’ access and willingness to attend the CRC screening programme in Norway. As the interviews were conducted six to eleven months before the start of the CRC screening programme, the study explored factors that may be of relevance for access to a preventive health measure that was not yet implemented.

## Material and methods

### Theoretical frameworks

Transnationalism refers to economic, societal, cultural and political cross-border activities and practices, and how these influence and modify people’s sense of belonging to a place, change their aspirations, imaginations and decisions in everyday life, and influence their identity [24]. There is substantial migration of Polish people between Norway and Poland. There are multiple daily low-cost flights between the countries, facilitating travel for work, vacation, family reunification and transnational care provision. In addition, Polish immigrants in Norway get updates from the Polish diaspora through traditional and social media and keep in touch with family and friends in Poland through the internet. We used transnationalism as a framework in our attempt to understand how the flow of information, ideas, people, traditions and cultural beliefs between the countries affect Polish migrants.

Access to a universal preventive health measure is a core part of this study. Access is more complex than the physical availability of a service. If a service exists free of charge, it might still be inaccessible if the people it is intended for do not have trust in it, can’t understand the way the offer is communicated to them or if it’s offered in a way that compromises their values. Levesque’s defines accessibility along five dimensions; “approachability”, “acceptability”, “availability and accommodation”, “affordability” and “appropriateness” [25]. These dimensions interact with five corresponding abilities in order to allow access; ability to perceive, ability to seek, ability to reach, ability to pay and ability to engage.

### Study setting and participants

This study is based on qualitative interviews with ten Polish immigrants in Norway; seven women and three men (Table 1). The interviews were conducted during the period from June to November 2021, which was six to eleven months before the start of the Norwegian CRC screening programme.

**Table 1:** Overview of participants

	n (%)
Age	
Mean (median)	56.2 years (56 years)
Range	52–59 years
Norwegian language skills	
Good	4
Able to participate in basic conversation	3
Limited/none	3
Higher education	
Yes	5
No	5
Work status	
Yes	7
No	3
Resided in Norway for at least 5 years	
< 5 years	1
5–9 years	1
≥ 10 years	8

Participants were recruited in two ways. Some of the participants were recruited through the authors’ networks. The authors of this manuscript have extensive networks within migrant health through research, operational work and personal experience of belonging to migrant communities. Other participants were recruited through Moja Norwegia, an internet portal for Polish people in Norway.

We aimed for a convenience sample of participants with varied backgrounds with respect to gender, age, education, work, time in Norway, place of residence, Norwegian language skills and ties to the Norwegian-Polish community. Three of the participants lived in the greater Oslo area, while the remaining participants lived in other parts of Western, Southern and Eastern Norway. Three of the participants had a prior cancer diagnosis, of whom one had been treated for CRC.

We aimed to interview participants aged 50 to 60 years old. We wanted to interview people aged 50–54, as they either turn 55 in the next few years, and will thus receive offers for CRC screening in the first years of the screening programme, or belong to an age group (56–65) targeted by the CRC screening programme even though they were too old to receive offers for screening when the programme started.

### Qualitative interviews

We aimed to get an understanding from the viewpoint of the participants rather than from our preconceived understandings, and emphasized to the participants that we wanted a dialogue. Interviews were semi-structured. Our interview guide included open-ended questions, letting the participants introduce issues of relevance that could take the conversations in directions that we might not have thought of prior to the interviews.

Two researchers, SB and EC, conducted the interviews. SB is a Norwegian-born male who speaks fluent Norwegian and English, but not Polish. EC is a Polish-born woman living in Poland, who has lived in Norway for several years. EC speaks Polish, Norwegian and English. SB conducted four interviews in Norwegian, and EC conducted six interviews in Polish. The duration of the conversations varied between 30 to 60 min.

**Fig. 1:**
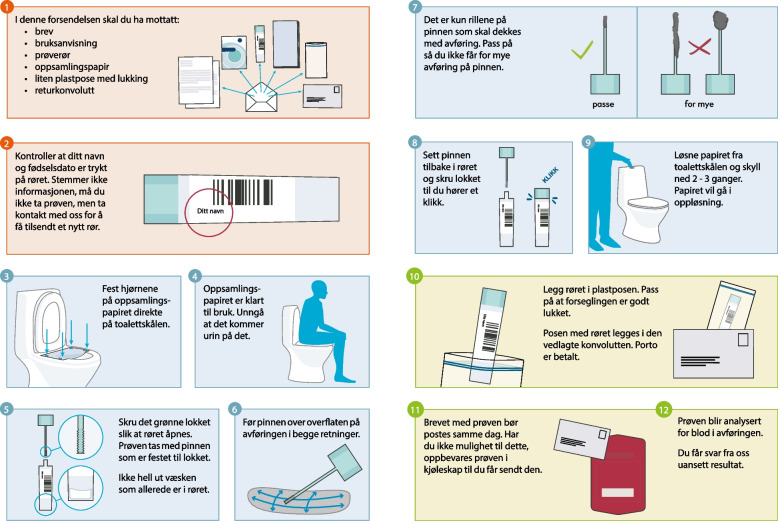
Feacal immunihistochemical test user manual Participants in the Norwegian colorectal cancer screening programme receive a user manual with illustrations and text together with the faecal immunohistochemical test. Figure 1 represents an early draft of the user manual

Due to the ongoing COVID-19 pandemic, we considered it inappropriate to meet participants in person. The interviews were thus conducted by phone, which enabled us to interview participants from across the country. The user manual that accompanies the FIT kit in the screening programme was sent to participants by email prior to the interview (Fig. 1).

### Data analysis

All interviews were written out by the interviewer. We translated transcripts in Norwegian sentence by sentence into English. Due to procurement rules, we were obliged to use the translation agency Tolkenett to translate interviews from Polish. Tolkenett could only translate from Polish to Norwegian, not from Polish to English. SB translated all ten interviews from Norwegian to English.

We performed thematic content analysis to understand CRC screening from the perspective of Polish immigrants. The translated transcripts were read several times, aiming to identify factors of relevance for understanding access to CRC screening. In readthroughs of the transcripts as well as during critical revision of the manuscript, we actively sought out factors of relevance to transnationalism, as well as to Levesque’s five dimensions of accessibility and their corresponding abilities.

For our initial codes, we aimed to identify a broad range of factors that could potentially be of relevance, aiming to identify as many factors as possible. Factors that concerned similar topics were grouped together in more comprehensive codes. The comprehensive codes were then grouped into initial themes. The initial themes finally resulted in the themes presented below, and the subthemes within the themes emerged from the comprehensive codes. The findings were continuously discussed between the authors as the study progressed, both through oral discussions and critical revision of the analyses and manuscript. Through revisions of the manuscript, both interviewers considered whether the presented findings were in keeping with data from the interviews.

All authors were part of the analytical process, which was lead by SB. SB and EC read through the transcripts and identified the initial codes. The remaining authors were involved once the initial codes were grouped into comprehensive codes. All authors gave feedback on the content, composition and interpretation of the categories from comprehensive codes to themes and subthemes, and through critical revision of the manuscript from the early drafts to the final version.

### Ethical considerations

The study was approved by the Norwegian Data Protection Authority at Oslo University Hospital (20/15902). Participants received consent letters by email. In keeping with the approval from the Norwegian Data Protection Authority, the interviewer ensured that the participants understood the content of the consent letter before orally giving an informed consent.

Transcripts of the interviews were stored on a secure server without directly person-identifying information. All participants were given pseudonyms, which are used in the presentation of findings below. Audio recordings were deleted as soon as the interviews were transcribed. The participants were informed about the right to have their information deleted before it was part of the formal analyses.

## Results

**Fig. 2:**
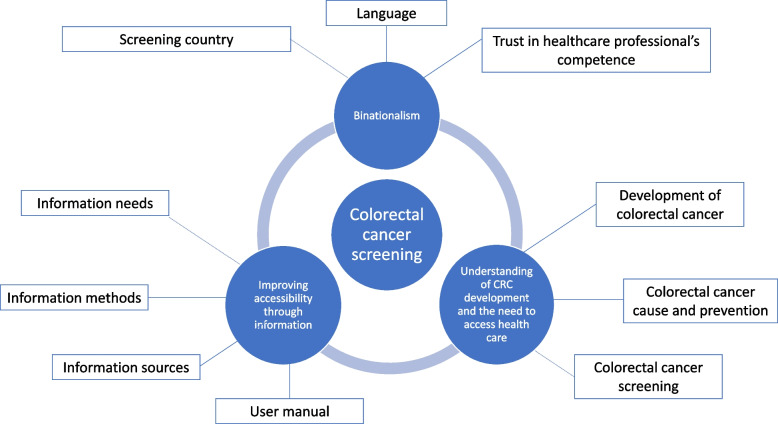
Overview of themes and subthemes Themes and subthemes of relevance for Polish immigrants’ access and willingness to attend the Norwegian colorectal cancer screening programme

We present our findings categorised into three themes and corresponding subthemes of relevance to Polish immigrants’ access and willingness to attend the Norwegian CRC screening programme (Fig. 2).

## First theme: understanding of colorectal cancer development and the need to access health care

### Development of CRC

The participants showed that they had knowledge about the development of CRC from polyps to cancer, and the development from localised, early-stage disease that can be cured by surgery, to late-stage, metastatic disease that is not available for curative treatment. They were, however, worried that early-stage CRC often does not give any symptoms. As described by David:

This is a really ugly cancer. You don’t get any problems in the beginning, and when it finally gives problems, it is very bad.

Participants had learned about symptoms that would make them worry about CRC from multiple sources, including relatives and friends, health care workers and media. The symptoms they were aware of included abdominal pain, difficulty passing stool, weight loss and blood in the stool. They emphasised the importance of being aware of such symptoms, which would make them approach a doctor, as illustrated by Ewa:

One has to check in the toilet what one leaves there. It’s not a nice sight, but it can help sometimes.

The knowledge that late-stage cancer could appear was something that worried participants. Some described that even the word cancer itself was scary and associated it with certain death. Others described how fear of CRC would result in feeling that an examination would be appropriate. Agnieszka suggested a way this fear could be used to convince people to approach an examination so that CRC could be detected at an early stage:

*It’s maybe a good idea to scare people with numbers and nasty images, like the way on cigarette packages. It should say that one might die from this type of cancer if it’s not detected early.*

### CRC cause and prevention

All participants mentioned diet or nutrition as a cause of CRC. Some offered specific types of food that could influence the risk of CRC and advised that one should eat less red meat and more vegetables and food with a high fibre content. Others offered more general advice about food, such as avoiding processed food, food that takes a long time to digest and food with lots of chemicals. Not only food, alcohol and smoking, but also other things that were ingested were suggested to cause CRC, as suggested by Agnieszka:

*Well, the environment is polluted. What we breathe in, what we eat, whatever enters our body [may cause CRC].*

Several participants suggested lack of physical activity and stress as causes of CRC. Others suggested that CRC might be hereditary or due to genetic predisposition. Most participants seemed to be aware of known risk factors for CRC.

The participants accepted that CRC should be prevented, and suggestions for how to prevent CRC were related to their knowledge about development and causes of CRC. Several participants discussed eating healthy, staying physically active, contacting their GP if symptoms appear and going for examinations as ways of preventing CRC.

### CRC screening

The participants differed greatly in their understanding of and experience with CRC screening. Most participants were aware of colonoscopy, and some had sought a colonoscopy themselves. Some participants were aware that colonoscopy can be performed in a screening setting, while others only knew of it as a diagnostic examination to investigate symptoms. The participants had a very positive attitude towards colonoscopy and accepted it as an appropriate examination, even if they had bad experience with colonoscopy themselves. Karolina elaborated on her experience with colonoscopy:

It was a tough experience for me. I experienced fear and pain. I told them I could not handle this pain. The examination was aborted, I don’t think it was performed correctly

*Interviewer: After all that, do you think it’s worth going for a colonoscopy?*

*Yes, of course it is*

Even though participants received the user manual that will accompany the FIT kit, the concept of FIT screening for CRC was unfamiliar for most of them. When asked specifically if they could think of other ways than colonoscopy to detect CRC, few volunteered FIT despite having read the user manual for FIT. The participants also seemed to trust colonoscopy more than FIT, as exemplified by Natalia:

*Maybe there is a difference in terms of stage. I don’t know whether the faecal test detects all stages, or whether one has to go for colonoscopy and the faecal test only detects the first phase.*

Some participants had given stool samples to their GPs to look for blood in the stool and deducted from their experience that if the sample revealed blood in the stool, then there was a need to investigate further with colonoscopy.

A few of the participants believed that they could be referred to FIT or colonoscopy through participating in the interview. We encouraged these participants to contact their GP if they had concerns about their colon health. Piotr volunteered to participate in the interview as he was curious about CRC screening as his GP in Norway had informed him that in Norway one does not refer patients for unnecessary examinations to look for diseases that do not present with symptoms:

*The Norwegian doctor told me that one doesn’t try to examine patients in advance [of symptoms]. This made me extra motivated to talk to you. Besides I take these examinations and know how important it is to participate in them.*

## Second theme: binationalism

### Language

The participants differed in their Norwegian language skills. Some of the participants did not speak Norwegian at all, while others spoke Norwegian well after living in Norway for decades. Most participants welcomed the idea of making screening more approachable by translating information letters about CRC screening to Polish, or perhaps having the information available in Polish online on the Norwegian Labour and Welfare Administration’s webpage (nav.no) or on the national public portal for health services (helsenorge.no). Piotr argued that he would probably not read information about CRC screening if he received it in Norwegian, but that he would at least read a few headings if it was written in Polish.

While the information letter and instructions are ways of one-way communication, several participants brought up an affordable aspect that increased appropriateness of services; that one has the right to have a qualified interpreter without any cost when communicating with public health services [26]. Others preferred having family members translate rather than having a professional interpreter who they did not know. Natalia summarized well how language issues could both aid and complicate communication:

*It depends on what situation you are in. If it’s just information, one can get it translated. But if there’s a need for dialogue, then language becomes a problem. If there’s an interpreter who translates purely mechanically… The relational part is important for language. And when the interpreter leaves, it’s family members who take care of you.*

### Screening country

In 2012, the countrywide Polish Colonoscopy Screening Programme replaced an opportunistic screening programme implemented in 2000 [27]. Several of the participants had undergone colonoscopy in Poland, both as a screening examination and as a diagnostic examination. Some of the participants had undergone diagnostic colonoscopy in Norway.

Through conversations, it became clear that many of the participants underwent examinations that were available and affordable in Norway when travelling to Poland, including screening examinations. While the user fee for hospital visits in Norway is about €40, participants paid €125–200 for colonoscopy in Poland. The fee in Poland included general anaesthesia, which is not routinely administered with colonoscopy in Norway. Despite being more expensive, having colonoscopy in Poland increased accessibility for some participants in terms of approachability, acceptability, and appropriateness, as they could receive the examination under general anaesthesia, as well as the staff speaking Polish and the participants having the opportunity to choose screening modality and treatment facility.

The participants differed in their opinion whether it was right to travel to Poland for examinations. Maria was reluctant to travel to Poland for screening examinations:

*I don’t think that’s the right attitude [to travel to Poland for examinations]. It’s ridiculous. If we stay here most of the year, we should function in this health system.*

For other participants it was important to have the opportunity to travel to Poland for colonoscopy. Mateusz’s wife travelled to Poland for colonoscopy, and he argued that one *should* travel to “the home country” for such examinations even when living in Norway. Ewa had undergone colonoscopy in Poland herself, and argued that she should be free to choose to have colonoscopy in Poland:

*What’s the problem? It’s a disease that develops over time, so even if there is a need for treatment one has time to travel back to Poland.*

### Trust in health personnel’s competence

The participants differed in acceptance of Norwegian health care professionals’ competence. To a large degree it seemed that participants with shorter duration of residence in Norway, poor Norwegian language skills or lack of knowledge about Norwegian public health services were discouraged from seeking health care in Norway. Often approachability was reduced due to own negative experiences with health services in Norway, or stories about other people with bad experiences. Some argued that doctors in Poland have more knowledge than doctors in Norway, as stated by Agnieszka:

*Norwegian doctors need to check all diseases, they google when patients are at doctor’s appointments, while Polish doctors have everything in their head. Polish doctors are better, they are more experienced.*

Several participants were reluctant to attend Norwegian doctors, arguing that Polish doctors dig more into medical problems, send more referrals for examinations and prescribe more medications. As described by Kasia:

*In Poland they send us very quickly for examinations. In Norway we often just get paracetamol tablets when we have some symptoms, so there is a huge difference between here and Poland.*

Piotr reflected on the different approaches experienced by himself concerning health services in Norway and Poland:

*I find the strategy of not performing examinations “just in case” interesting. I have not lived here for so long, but I see that it’s a healthy society, so it’s dawned on me that this approach works in some way or the other.*

Other participants, often those who had lived in Norway for many years, had a negative attitude towards health care professionals in Poland and believed that they got better services in Norway.

Trust was a complex issue that influenced the participants’ health-seeking behaviour in a variety of ways. Participants described Polish people as being sceptical. David argued that Polish people have less, if any, trust in doctors, and bring this lack of trust with them to Norway. Natalia elaborated:

*If someone talks in favour of public services, Polish people might say that perhaps they are working for them. They will double check and triple check, and you don’t know what sources they check.*

While participants described scepticism towards doctors as in issue both in Poland and Norway, several participants offered trust in Polish doctors as a reason for why it was acceptable to travel to Poland for colonoscopy. One participant, who did not trust doctors in Norway, argued that everyone, not only Polish people, are afraid of getting a wrong diagnosis from Norwegian doctors.

Some of the participants even a had Polish GP in Norway, stating that they had more trust in Polish doctors than in Norwegian doctors. However, participants with Polish GPs were also critical towards their GP. A participant who was dissatisfied with his Polish-speaking GP in Norway offered the following explanation for why he did not change GP:

*Well, because he speaks Polish*

Other participants, including most of those who had lived in Norway for a long period of time, described a high degree of trust that facilitated accessibility to Norwegian health services. These participants also encouraged other Polish immigrants to attend health services in Norway rather than travelling to Poland. Participants who trusted Norwegian health services seemed to argue that lack of trust was associated with a core aspect of the Polish identity, arguing for instance that complaining reflects a Polish trait, and that Polish people criticise everything without seeing the good things that Norway offer them.

## Third theme: improving accessibility through information

### Information needs

In order to make CRC screening more available, acceptable and approachable, participants articulated a need for basic information about CRC and CRC screening. Some argued that accessibility to CRC screening for other Polish people than themselves might be hindered by lack of information or education. In terms of CRC, the information they requested included who was at risk of getting disease, the symptoms of CRC and what they could do to avoid getting CRC. In terms of CRC screening, the information they requested included how the test is performed, what happens to the test after the examination and the risks associated with not having CRC screening. Agnieszka emphasised the importance of telling people the purpose of CRC screening, as her experience was that health care professionals sometimes don’t explain what they are looking for when conducting examinations. None of the participants requested information about negative effects of screening.

Not all participants saw the need for more information about CRC or CRC screening. Some argued that they had gathered all the information they needed, for instance through relatives or friends who had been diagnosed with CRC or who had received a colonoscopy for other reasons. Others argued that they could find available and appropriate information themselves if needed, as exemplified by Ewa:

*I am very curious, and for everything I need to know I seek information for myself. I don’t think there is more I’d like to know.*

### Information methods

The CRC screening programme in Norway offers screening by sending a FIT kit with accompanying information about CRC screening and a user manual written in Norwegian by post. Agnieszka argued that letters are an affordable way of distributing information to a large number of people who will likely read it, but she was unsure whether sending letters was sufficient to increase accessibility:

*It’s not always enough to send a letter. It is very easy to do what I did with the mammography appointment, that one just puts it aside, and then it’s not read.*

While the FIT kit itself cannot be sent digitally, several participants argued that accompanying information about the test should be sent digitally, either by email or through helsenorge.no. Some participants seemed to find digital information accommodating, while Karolina had a more idealistic reason for requesting digital information, arguing that digital information was more environmentally friendly as it saves paper.

### Information sources

Other than information from the service provider, the Cancer Registry of Norway, the participants offered several sources from which they would seek information about CRC and CRC screening. The participants encouraged increasing approachability by providing information through internet portals for people with a Polish background in Norway, including “Moja Norwegia”, from which six of the participants were recruited, wataha.no and the Facebook-group “Polakker i Oslo” (translated from Norwegian: “Poles in Oslo”). Agnieszka was worried that there was both correct and incorrect information online, and Piotr expressed some concerns with internet forums:

*I have read many opinionated, one-sided remarks from people complaining about Norwegian health services. They don’t realise that they have communication problems themselves.*

Several participants suggested that informing about CRC screening through traditional media, including national television and editor-controlled online newspapers, could increase accessibility, and Julia explained that people in Poland are encouraged to go for CRC screening through advertisements on TV. When asked whether Polish people in Norway read Norwegian online newspapers, she replied that they don’t, but that they do watch TV.

Participants explained how they got information about CRC and screening through family members and friends. Some of the participants did not speak Norwegian and did not have any Norwegian friends, and thus had limited possibilities to seek and reach information from Norwegian public sources. Kasia described how it was natural to ask family members or friends who had suffered from CRC. Some of the participants described cancer as taboo, as Karolina explained:

*Such things were not discussed within our family. Later, when I was older, I got to know that my uncle had CRC.*

Several of the participants requested that the GP should be engaged in informing them about CRC screening. Some suggested that the GP could send information, as the GP would know whether the information needed to be translated to Polish. Others suggested providing information at the GP’s office, for instance in the form of brochures.

The participants also reported getting information about CRC and CRC screening from Google and YouTube.

### User manual

Participants received the user manual for the FIT kit prior to the interview. The user manual consists of a step-by-step instruction with images and text (Fig. 1). While many participants did not seem to understand that FIT was a CRC screening method, they found the format of the user manual acceptable and appropriate, as explained by Natalia:

*It was very detailed, and the images were appropriate. In that way you can’t go wrong.*

The participants argued that the brochure gave a simple and clear message that would allow people to understand how to use the FIT kit. Piotr, who did not speak Norwegian, stated:

*It has pretty obvious iconography. It took me a minute. These images are so obvious.*

Some of the participants had translated the information. Karolina had understood most of the information from the images, but there was also some information that she only understood after translating the text from Norwegian to Polish. Ewa also had to turn on a translator to make the information more approachable, and expressed:

*One may get a hint of what it is about from the images. However, I had to turn on a translator to understand the text that accompanied the images. It would be better if it was all written in Polish. Then one would not have to spend time translating, and one could be sure that one did not misunderstand.*

## Discussion

In this study based on qualitative interviews of ten Polish immigrants, we identified several interlinked factors that could potentially influence access to the CRC screening programme in Norway. With basis in transnationalism and accessibility as frameworks, we grouped the factors identified into three themes; understanding of CRC development and the need to access health care, binationalism and improving accessibility through information.

As the Norwegian CRC screening programme has only recently started, there have not been any qualitative studies exploring CRC screening uptake among immigrants in Norway. A master’s degree study explored non-immigrant men’s experiences in the pilot study preceding the national CRC screening programme [28]. The study highlighted the GP as an important advisor in terms of facilitating an informed decision regarding screening participation, which is in line with the men and women in our study, who described the GP as a key person in terms of health seeking behaviour.

Some observations from studies from other countries resonate well with our findings, such as that appropriate awareness of CRC screening, positive attitudes for CRC screening and having a close relative or friend diagnosed with CRC facilitate CRC screening, while language barriers may prevent screening [11]. Other observations in our study seem to differ from other studies. While fear of cancer has been identified as a barrier against screening in other studies [11], it rather appeared to facilitate screening for our participants. Low income has been associated with non-uptake of CRC screening [10], but the cost of the examination did not appear to be an issue for our participants.

The participants in our study represented a heterogenous group in terms of education, work, time in Norway, Norwegian language skills, ties to the Norwegian-Polish community and lived experience of cancer. The interviews revealed a myriad of perspectives, including congruent and diametrically opposing views. The themes and subthemes appeared to be interlinked. For instance, information sources appeared to be of influence for the participants’ trust in health care professionals’ competence as well as for knowledge about CRC and CRC screening. The participants seemed to be concerned about CRC, in favour of CRC screening and eager to have or seek knowledge about CRC screening. Many participants preferred Polish GPs. Our findings are consistent with a study with focus groups with 15 Polish immigrants in Norway that revealed that Polish immigrants want information about cancer prevention, have a fear of cancer and want information about Polish doctors in Norway or doctors with education from Poland [23].

Immigrants’ expectations towards health services in their country of residence are shaped by cultural health beliefs and experiences with health services in their country of birth [29]. For many Polish immigrants, the health care system in Poland is a reference point to which they compare services [30]. Immigrant GPs have themselves a personal experience of being an immigrant and may develop cultural awareness that could put them in a position where they better understand the lived lives of immigrants with similar backgrounds, and thereby perhaps helping immigrant patients better than non-immigrant GPs [31]. Polish GPs do not only speak Polish but are also well positioned to consider social and cultural aspects that are not immediately available for someone who has grown up outside Poland. This could explain why some of the participants had Polish GPs, despite being dissatisfied with the GP’s service. Participants’ knowledge and experience from Poland of a screening programme using colonoscopy, the possibility for general anaesthesia and a different way of communication between doctors and patients could contribute to some Polish immigrants not having their needs met in the Norwegian health care system, thus considering health care services in Poland as more accessible than in Norway.

The request to involve the GP for information could be independent of patients’ gender, country of origin and screening programme. Many of our participants valued the GP as someone who could advise them about health-related issues, regardless of whether the GP spoke Polish or not. In qualitative interviews from Norway, immigrant women from Pakistan and Somalia targeted for cervical cancer screening and immigrant women from Pakistan targeted for breast cancer screening requested that the GP should be more involved in informing about screening examinations [32, 33]. The GP service in Norway, which aims to provide everyone with a permanent doctor over time, is a public health service with high user satisfaction [34]. The GP service is, however, under great pressure, with an increasing workload, insufficient funding and problems recruiting doctors [35]. We believe that GPs are in an excellent position to provide information about CRC screening and other preventive health care measures to their immigrant patients. However, increasing their workload could contribute to worsen the so-called “GP crisis”.

Several participants argued that Polish doctors prescribe more medications and send more referrals than Norwegian doctors, and even found the attitude of not looking for asymptomatic disease odd. “Choosing wisely” is an international campaign targeting doctors and patients with an aim to reduce diagnostics and treatment that do not add value, arguing that these lead to overtreatment, have no benefit and may cause harm [36]. For instance, while participants in our study mentioned the possibility for general anaesthesia as a reason for having colonoscopy in Poland, one could argue that general anaesthesia puts the patients at unnecessary risk as it is not necessary in order to perform colonoscopy and involves risks, including an infrequent risk of death. The choosing wisely campaign has gained momentum in Norway, and the Norwegian Medical Association argues that the campaign should increase its focus on patients [37]. While the campaign intends to benefit patients, our participants perceived that withholding tests and treatment that the doctor deemed unnecessary decreased accessibility. In this regard, Polish people are representative for other migrant groups [29], and the finding highlights the need for specific training of health care professionals in cultural competence when presenting options for diagnosis and treatment for an increasingly diverse population.

Nationwide public health measures aim to be accessible for the entire target population. We anticipate that the CRC screening programme with FIT will be affordable, as it is free of cost and can be performed at a time and place that suits the participants. However, our findings show that there might be issues with the other dimensions of accessibility. Language difficulties, a preference for Polish health care professionals and issues related to information needs, methods and sources were among factors identified that could limit Polish immigrants’ accessibility to CRC screening.

From the service provider’s perspective, low uptake among subgroups is a problem in a screening programme that should be universally accessible. However, in terms of CRC screening among immigrants from Poland, the issue is more complex than simply considering the proportion of people who go through with FIT in the CRC screening programme. The ease of movement between Norway and Poland facilitates availability and accommodation of screening in Poland, and the possibility to choose to undergo CRC screening in Poland seemed important for some participants’ autonomy. Low uptake in the Norwegian CRC screening programme will not itself mean that immigrants from Poland are underscreened, as they might have had screening in Poland instead of Norway. Further, CRC screening in Poland is offered as colonoscopy, which means screening is offered with a superior method in Poland until colonoscopy is available in the Norwegian CRC screening programme.

Several measures can be taken to increase accessibility to CRC screening for Polish immigrants in Norway. As colonoscopy replaces FIT as screening method in Norway, the technical quality of screening will no longer be superior in Poland. Language barriers are important determinants of health literacy, health care access and utilization and adherence to care [12, 38]. Approachability and acceptability of CRC screening may be increased by translating information to Polish and using health care professionals to inform about CRC screening. This may include providing information in Polish through internet portals, GPs and Polish-speaking health care professionals. While the right to have an interpreter does not concern the written information participants receive about FIT, an interpreter could increase appropriateness and acceptability of colonoscopy, whether as a follow-up examination after a positive FIT or when the screening programme implements colonoscopy. The instructions that accompany the FIT kit appeared to facilitate availability of CRC screening. However, when colonoscopy is introduced as a screening method, transport and lack of social support could be among factors limiting ability to reach the screening examination. Affordability did not seem to be an issue for the participants in our study. While CRC screening with FIT does not have any monetary costs, some of our participants paid for flights and examination-related costs in Poland. We do not know whether these participants travelled to Poland for an examination, or if they had the examination in Poland as they were travelling for other reasons. When colonoscopy becomes available in the Norwegian CRC screening programme, the user fee for colonoscopy, which is about €40, could limit affordability for someone who is not able to prioritise money for the examination. In terms of appropriateness, our study revealed that there is a need to inform participants that FIT is a screening method that has been shown to reduce CRC mortality, as many participants did not seem to understand that FIT was a screening method despite reading the user manual.

### Limitations

While this study gives valuable insight when planning how to inform Polish immigrants in Norway about CRC screening, the study has some limitations. Several factors may limit the credibility of our findings. A high proportion of our participants (30%) had a prior cancer diagnosis, which could result in a population that were more in favour of cancer screening than Polish immigrants in general. However, only one participant had a prior CRC diagnosis, and both participants with and without a prior cancer diagnosis appeared to encourage health-seeking behaviour and to be in favour of CRC screening. Most of our participants had lived in Norway for at least ten years, and we could thus expect that our participants were more familiar with Norwegian health services than recent immigrants. This limits our possibility to explore how length of stay and a feeling of being new in a country shapes participation. The number of participants may also limit the credibility of the study. Initially, we planned to interview at least twelve participants. The ten participants interviewed represented very diverse backgrounds and contributed with a wide range of perspectives, and the last few interviews largely built on perspectives from the previous interviews. We thus considered that we had reached data saturation. The credibility of our study could be increased by including more recent immigrants and participants who were not in favour of CRC screening. We do, however, believe our participants represent a heterogenous group in terms of socioeconomic factors and have contributed with a wide range of perspectives, and that Polish immigrants who were not part of this study will consider our findings recognisable.

In terms of transferability, some of our findings can be useful when considering preventive health care measures for other immigrant groups and for immigrants in other countries. Other findings must be interpreted with caution. While Polish immigrants may undergo screening examinations in Poland, it is less likely that other populous immigrant groups in Norway, such as immigrants from Somalia and Pakistan, attend screening examinations in their country of birth.

By conducting interviews by phone, we were able to recruit participants from across the country and to talk to them without breaching COVID-19-restrictions. However, we missed out on important non-verbal communication. In retrospect, this limitation could have been reduced with video calls.

A final limitation we want to highlight is the different languages used in this study. All interviews were translated from Norwegian to English. Due to procurement rules, six of the interviews had to be translated from Polish to Norwegian before we could translate them to English. SB and EC’s critical revision of the analyses and manuscript reduced the possibility that the participants’ opinions got misunderstood in translation.

## Conclusion

In conclusion, in a transnational setting with substantial migration between Poland and Norway, we identified several factors that could influence Polish immigrants’ accessibility to the Norwegian CRC screening programme. In order to reduce morbidity and mortality from CRC, measures to improve accessibility for Polish immigrants should target these factors. Such measures include increasing cultural competence among health care providers and providing information in Polish through Polish-speaking health care professionals, GPs and internet portals used by the Polish-speaking community in Norway. The findings in our study are of relevance when policy makers and service providers plan how to inform immigrant groups about current or proposed preventive health measures.

## Data Availability

Data sharing is not applicable to this article as no quantitative datasets were generated or analysed during the current study. Qualitative data generated from this study are included in this published article.

## Abbreviations

CRC: Colorectal cancer
FIT: Faecal immunochemical test
GP: General practitioner
